# Authors’ Reply to: Using Caution When Interpreting Gender-Based Relative Risk. Comment on “The Effect of Cardiovascular Comorbidities on Women Compared to Men: Longitudinal Retrospective Analysis”

**DOI:** 10.2196/36801

**Published:** 2022-03-25

**Authors:** Elma Dervic, Carola Deischinger, Nina Haug, Michael Leutner, Alexandra Kautzky-Willer, Peter Klimek

**Affiliations:** 1 Section for Science of Complex Systems Center for Medical Statistics, Informatics and Intelligent Systems Medical University of Vienna Vienna Austria; 2 Complexity Science Hub Vienna Vienna Austria; 3 Department of Medicine III Clinical Division of Endocrinology and Metabolism, Gender Medicine Unit Medical University of Vienna Vienna Austria; 4 Gender Institute Gars am Kamp Austria

**Keywords:** gender gap, sex differences, cardiovascular diseases, acute myocardial infarction, chronic ischemic heart disease, gender, diabetes, smoking, risk factors, comorbidities, relative risk, interaction

We thank Janszky [1] for their time and observations on our paper [2]. We appreciate the comments.

Our analysis was done on a large data set of hospital diagnoses from 1997 to 2014. We developed a systematic approach to detect all significant gender differences across all comorbidities associated with cardiovascular disease (CVD). In our paper [2], we reported all risk factors and calculated sex differences as a measure of differences using logarithmic odds ratios between male and female patients in units of pooled standard errors. As a limitation, we pointed out that we cannot rule out specific unobserved confounders as well as the limitations of our in-hospital data set. We thank Janszky [1] for providing an illustrative example of how such a confounding influence could work.

It is clear that correlation is not causation, and we did not make any statement on causality. We analyzed the order of diagnoses by conducting a “time directionality analysis,” and our results showed us which diagnoses were “typically diagnosed before.” In the *Limitations* section, we emphasized this as well: “Given the purely observational nature of our dataset, no statements on causality can be made based on this analysis.” Janszky’s [1] comment clearly shows why it is important to repeatedly stress such limitations.

The motivation behind our work is to increase awareness of the need for gender-specific medicine. It has been well described that the female sex overall is protective in the development of CVD due to biological and psychosocial factors but that metabolic diseases like diabetes attenuate this protective effect [3]. Yet, our knowledge on potential sex-dimorphic pathophysiological mechanisms remains limited, in particular in relation to CVD. With our work, we aim to show how observational data can be used to rapidly generate hypotheses regarding sex differences in disease risk at scale and thereby initiate further research that aims to clarify their potential causal mechanisms.

## Abbreviations

CVD: cardiovascular disease
